# Synthesis of eco-friendly layered double hydroxide and nanoemulsion for jasmine and peppermint oils and their larvicidal activities against *Culex pipiens* Linnaeus

**DOI:** 10.1038/s41598-024-56802-y

**Published:** 2024-03-22

**Authors:** Ibrahim Taha Radwan, Hanem F. Khater, Shaimaa H. Mohammed, Abdelwahab Khalil, Mohamed A. Farghali, Mohammed G. Mahmoud, Abdelfattah Selim, Eman A. Manaa, Noha Bagato, Mohamed M. Baz

**Affiliations:** 1 Supplementary General Sciences Department, Faculty of Oral and Dental Medicine, Future University in Egypt, Cairo, 11835 Egypt; 2 Department of Parasitology, Faculty of Veterinary Medicine, Benha University, Toukh, 13736 Egypt; 3 Zoology and Entomology Department, Faculty of Science, Al-Azhar, University (Girls Branch), Cairo, Egypt; 4 Entomology Division, Zoology Department, Faculty of Science, Beni-Suef University, Beni -Suef, 62521 Egypt; 5 Nanotechnology and Advanced Materials Central Lab (NAMCL), Regional Center for Food & Feed (RCFF), Agricultural Research Center (ARC), Giza, Egypt; 6 Plant Protection Department, Faculty of Agriculture, Zagazig University, Zagazig, Egypt; 7 Department of Animal Medicine (Infectious Diseases), Faculty of Veterinary Medicine, Benha University, Toukh, 13736 Egypt; 8 Animal and Poultry Production, Department of Animal Wealth Development, Faculty of Veterinary Medicine, Benha University, Toukh, 13736 Egypt; 9 Egyptian Petroleum Research Institute (EPRI), PO Box 11727, Nasr City, Cairo, Egypt; 10 Departments of Entomology, Faculty of Science, Benha University, Benha, 13518 Egypt

**Keywords:** *Jasminum sambac*, Oil–water nanoemulsion, Essential oil drug release, *Mentha arvensis*, Biochemistry, Biological techniques, Plant sciences, Zoology

## Abstract

Mosquito-borne diseases represent a growing health challenge over time. Numerous potential phytochemicals are target-specific, biodegradable, and eco-friendly. The larvicidal activity of essential oils, a jasmine blend consisting of Jasmine oil and Azores jasmine (AJ) (*Jasminum sambac* and *Jasminum azoricum*) and peppermint (PP) *Mentha arvensis* and their nanoformulations against 2nd and 4th instar larvae of *Culex pipiens*, was evaluated after subjecting to different concentrations (62.5, 125, 250, 500, 1000, and 2000 ppm). Two forms of phase-different nanodelivery systems of layered double hydroxide LDH and oil/water nanoemulsions were formulated. The synthesized nanoemulsions showed particle sizes of 199 and 333 nm for AJ-NE and PP-NE, with a polydispersity index of 0.249 and 0.198, respectively. Chemical and physiochemical analysis of TEM, SEM, XRD, zeta potential, drug loading capacity, and drug release measurements were done to confirm the synthesis and loading efficiencies of essential oils' active ingredients. At high concentrations of AJ and PP nanoemulsions (2000 ppm), O/W nanoemulsions showed higher larval mortality than both LDH conjugates and crude oils. The mortality rate reached 100% for 2nd and 4th instar larvae. The relative toxicities revealed that PP nanoemulsion (MA-NE) was the most effective larvicide, followed by AJ nanoemulsion (AJ-NE). There was a significant increase in defensive enzymes, phenoloxidase, and α and β-esterase enzymes in the treated groups. After treatment of L4 with AJ, AJ-NE, PP, and PP-NE, the levels of phenoloxidase were 545.67, 731.00, 700.00, and 799.67 u/mg, respectively, compared with control 669.67 u/mg. The activity levels of α-esterase were 9.71, 10.32, 8.91, and 10.55 mg α-naphthol/min/mg protein, respectively. It could be concluded that the AJ-NE and PP-NE nanoformulations have promising larvicidal activity and could act as safe and effective alternatives to chemical insecticides.

## Introduction

Arthopode-borne diseases represent a growing health challenge over time^1–10^, especially after global changes^2^, as over 40% of the world’s population is threatened by mosquito-borne diseases^11^, causing health problems, social disorders, economic losses, and millions of deaths annually^12^. *Culex pipiens* acts as a vector of filariasis and many arboviruses such as the West Nile virus and the Rift Valley fever virus^13–18^. Mosquitoes are currently controlled through synthetic insecticides and repellents^19,20^ inducing negative health and environmental implications^21,22^; therefore, searching for more environmentally acceptable pesticides is an imperative demand^23–28^**.**

Plant extracts or oils have long been used for medicinal purposes and fighting against pests^1,29–31^**.** It has been noted that the low-risk phytochemicals have been identified as potential biological control agents such as parasiticides^31–40^ and insecticides, acting as ovicides, adulticides^41–46^**,** larvicides^43,47,48^, growth regulators^42,49–52^, deterrents, and repellents^42,53,54^. Therefore, there has been a growing interest in using plant extracts and essential oils as alternatives to synthetic pesticides^42,47,55–58^**.** Despite their pesticidal efficacy, botanical pesticides was adversely affected by their low physicochemical stability, high volatility, thermal breakdown, and low water solubility^43,59,60^.

Natural pesticides, especially those converted into nanoformulations, will undoubtedly be environmentally friendly^61,62^, effective and economical tools against insect pests^24,27,52,63–67^. When insects are exposed to pesticides, they begin to resist them through the metabolic process to mitigate their damage. The elevation of the detoxification enzymes in the insect’s body is an indication of the efficiency of the used pesticide^68,69^.

Over the past years, lipid-based nanoparticles like nanoemulsions, microemulsions, solid lipid NPs, nanostructured lipid carriers, exosomes, and liposomes have attracted much interest in delivery systems due to their unique properties of preparation, transparency, and long-term stability^70^. Nanoemulsions have been extensively studied as a reason for their potential applications in various industrial fields, such as food, cosmetics, pharmacology, and pesticide formulations^71,72^. Nanoemulsions are considered intermediates between classical emulsions and nanoemulsions are usually in the–20—500 nm size range^73–75^. The small size of the nanoemulsion dispersion facilitates penetration or absorption by the cells^76^. The main categories of nanoemulsions are oil-in-water or water-in-oil, transparent, translucent, or colloidal dispersions^75,77,78^. Oil-in-water emulsions mainly contain the lipophilic bioactive segment (essential oils or any oil) representing the oil part and an aqueous medium in the presence of an emulsifier like Tween 20 as a non-ionic surfactant^79,80^. Nanoemulsion droplet size depends on the component composition and the homogenization method.

One important class of inorganic lamellar nanomaterials is Layered Double Hydroxides (LDH) representing solid-phase adsorbents of positively charged brucite-type layers of mixed metal hydroxides. The positively charged metal oxide is surrounded by multiple sheets of hydroxide anions with interpolated anions and water molecules. There are different examples of LDH such as MgAl LDH, CoAl LDH, and ZnAl LDH. They have the same stacked layers [M(II)(OH)_2_] containing some of its divalent cations like Mg^2+^, Co^2+^, or Zn^2+^ substituted by trivalent cations Al^3+^ or Fe^+3^ at the centers of the octahedral sites of the hydroxide sheet, whose vertex contains hydroxide anions that are distributed by three octahedral cations and pointed toward the interlayer region. Regarding the formula [[(M^*II*^)_*1-x*_(M^*III*^)_*x*_(OH)_*2*_]^*x*+^(A^*n−*^_*x/n*_) *m*H_*2*_O], M^2+^ and M^3+^ are divalent and trivalent cations, such as Mg^2+^, Zn^2+^, Ni^2+^, and Co^2+^; while the trivalent cations were represented as Al^3+^, Cr^3+^, and Fe^3+^, respectively. Owing to the partial substitutions of M^3+^ for M^2+^, the LDH sheets are positively charged and need to be neutralized by the intercalation of anions (An −), like NO^3−^, Cl^−^, CO_3_^2−^or SO_4_^2−^ whereas the x value is calculated by the ratio MIII/MII + MIII and it is usually between 0.17 and 0.33; however, also higher x values were reported^81–84^.

Different nanocarriers like polymeric, metal and metal oxides, and lipid nanoformulations have been investigated to produce acceptable delivery applications for essential oils by enhancing their stability properties as insecticides^24,25,27,28,85–87^. Nanoscience and nanotechnology present new alternatives to conventional pesticides with better physicochemical characteristics of the nanomaterials, improving their bioavailability in different ways for the target organisms. Therefore, nanotechnology has been used in the formulation of many new nanometric products, including nanoparticles, nanofibres, nanoemulsions, and nanocapsules in different fields^88,89^. The present work aimed to synthesize two forms of phase-different nanodelivery systems of layered double hydroxide LDH and (Oil/Water) nanoemulsion loaded with essential oils, a Jasmin oil blend (Jasmine oil and Azores jasmine) and peppermint, and to compare their efficacies before and after nanoformulations against 2nd and 4th larval instars of *Cx. pipiens* to present an effective insecticidal agent based on greener and safer components.

## Materials and methods

### Chemistry

Cobalt nitrate (CoNO_3_.6H_2_O), aluminum nitrate (AlNO_3_.9H_2_O), Zinc nitrate (ZnNO_3_.6H_2_O) bi-distilled water, urea, citric acid, trisodium salt, butyl alcohol, sodium hydroxide (NaOH), sodium nitrate (NaNO_3_), tween 20, sodium glycocholate, and oleic acid were purchased from Alfa Aesar, Germany. Essential oils of a commercially available jasmine oil blend consisting of Jasmine oil and Azores jasmine (*J. sambac* and *J. azoricum,* respectively*)* (Oleaceae) and peppermint (*M. arvensis*) (Lamiaceae) were bought from “Cap Pharm” EL CAPTAIN *Company* for extracting natural oils, plants, and cosmetics (El Obor, Cairo, Egypt); we would refer to the used oils as AJ and PP, respectively. All chemicals and essential oils were used without further purification or distillation.

### Synthesis of solid phase nanocarrier

#### Hierarchical three-dimensional Co-AlLDH

Hierarchical three-dimensional CoAl-LDH was synthesized via urea hydrolysis^90^ Co LDH. Exactly 0.698 g of cobalt nitrate hexahydrate Co(NO_3_)_2_.6H_2_O and 0.3 g of aluminum nitrate nonahydrate Al(NO_3_)_3_ 9H_2_O were mixed and dissolved in 40 ml bi-distilled water and 40 ml butyl alcohol with swirling for five min followed by stirring the mixture for 30 min. A proper amount of urea (0.384 g) as an exfoliating agent and 15 mg of citric acid trisodium salt were added, and the stirring was completed for 45 min. Next, the mixture was collected in a Teflon-lined stainless steel autoclave reactor to be thermally treated at 120 °C for 12 h. After heating completion, the reactor was allowed to cool to the room temperature, and the purple slurry was collected by filtration (12,000 rpm for 10 min). The filtrate was removed, and bi-distilled water was replaced several times to achieve perfect washing to get rid of unreacted salts, finally, washed three times with ethanol, then dried at 60 °C for 48 h to obtain a three-dimensional Co LDH. The abbreviation of CoAl LDH (MA) was used for peppermint oil delivery and CoAl LDH (AJ) was used for jasmine oil blend (AJ) delivery on CoAl LDH nanocarrier.

#### Synthesis of Zn-Al LDH

Synthesis of ZnAl LDH was done using the co-precipitation^56,61,91^ method as follows: a solution mixture of zinc nitrate hexahydrate Zn(NO_3_)_2_.6H_2_O (3.33 g) and aluminum nitrate nonahydrate Al(NO_3_)_3_.9H_2_O (1.39 g) were dissolved in 40 ml distilled water, then added to 100 ml of well-stirred solution mixture of sodium hydroxide (2 M) and sodium nitrate (0.2 M) to enhance the chance of the formation of a nitrate ion as a counter ion and the stirring was completed for 4 h. The resulting green gel-like layer was separated via centrifugation to remove water and unreacted salts. After washing several times with bi-distilled water and separation through centrifugation at 12,000 rpm for 10 min, the slurry was hydrothermally treated in a Teflon-lined stainless steel autoclave reactor at 80 °C for 24 h to improve its crystallinity and crystal shape. After cooling to room temperature, the green slurry was collected by centrifugation (12,000 rpm for 10 min) and washed three times with ethanol, then dried at 60 °C for 48 h. The abbreviation of ZnAl LDH (MA) was used for Peppermint (PP) oil delivery, and ZnAl LDH (AJ) was used for Jasmine oil blend (JA) oil delivery on the ZnAl LDH nanocarrier.

#### Drug loading of essential oil active ingredients on the LDH Nanoclays

The adsorption experiments of the active ingredients of both jasmine and peppermint oils onto CoAl LDH and ZnAl LDH were done at 25 °C using non-aqueous solvents as follows: 2 g of LDH (CoAl LDH or ZnAl LDH) was dispersed in organic solvent like ethyl acetate (EA), tetrahydrofuran (THF), or chloroform and placed in a three-neck round bottom flask, followed by degassing through nitrogen gas purging for 10 min. A well-mixed solution of 5 g of AJ or PP essential oils was dissolved in 15 ml of the same organic solvent (as AJ and PP essential oils are immiscible in water) and stirred gently for 8 h. The resulting slurry was centrifuged, washed with a small amount of the same solvent, and dried at room temperature under vacuum (to avoid breaking down volatile active ingredients). Each obtained material was ground gently in a porcelain mortar and stored in a glass bottle at a temperature below 10 °C.

#### Detection of drug released from loaded LDH Nanoclays

To verify whether LDH is suitable for the adsorption of the active ingredients of AJ and PP essential oils or not, 2 g of LDH-loaded essential oil was placed in a 100-ml beaker containing absolute ethanol (as a polar solvent to trap the nonvolatile polar compounds). To get the active ingredient released, mechanical stirring was applied for 3 h at room temperature. The same procedure was repeated using n hexane to detect the volatile non-polar compound if released, the solid slurry of LDH was collected by centrifugation and the supernatant was concentrated in each case and analyzed using GC/MS to determine nonpolar volatile active ingredients if released. The other portion was injected directly into LC–MS/MS, to determine the polar nonvolatile active ingredients, using liquid chromatography–electrospray ionization–tandem mass spectrometry (LC–ESI–MS/MS with an Exion LC AC system for separation and SCIEX Triple Quad 5500 + MS/MS system equipped with electrospray ionization (ESI) for detection).

The separation was performed using ZORBAX SB-C18 Column (4.6 × 100 mm, 1.8 µm). The mobile phases consisted of two eluents: A: 0.1% formic acid in water and B: acetonitrile (LC grade). The mobile phase was programmed as follows: 2% B from 0 to 1 min; 2–60% B from 1 to 21 min; 60% B from 21 to 25 min; and 2% B from 25.01 to 28 min. The flow rate was 0.8 ml/min, and the injection volume was 3 µl. For MRM (Multiple Reaction Monitoring, a highly sensitive and specific mass spectrometry technique selectively quantifies the compounds within complex mixtures) analysis of the selected polyphenols, positive and negative ionization modes were applied in the same run with the following parameters: curtain gas of 25 psi; Ion Spray voltage of 4500 and-4500 for positive and negative modes, respectively; source temperature of 400 °C; ion source gas 1 and 2 of 55 psi with a declustering potential of 50; collision energy of 25; and a collision energy spread of 10.

#### Synthesis of liquid-phase nanocarrier (Oil/water Nanoemulsion)

The synthesis protocol of AJ and PP essential oils loaded was carried out using the homogenization method as follows: in a 50-ml beaker (B1), 5 g of PP or AJ oils were warmed up to 37 °C^92–94^ using a water bath; another beaker (B2) contained 10 ml of distilled water, 0.5 g sodium glycocholate, 0.25 ml butanol as co-surfactants, and 4 ml tween 20 were mixed and wormed up to the same temperature. For oil/water nanoemulsion preparation, the two beakers were mixed using magnetic stirring for additional 5 min. then the final nannoemulsion was quenched rapidly with the addition of 35 ml ice-cold water with sonication for 10 min to obtain 50 ml of narrow-size distributed nanoemulsion. The final nanoemulsion was placed in a 50 ml plastic Falcon tube and kept under cold conditions (less than 10 °C). For nanoemulsion characterization, 1% Mannitol or sucrose solution was added to the dispersion as a cryoprotectant (10% v/v) to obtain a semi-solid substance through lyophilization for two days at -45 °C using a freeze dryer. The abbreviation PP-NE was used for Peppermint (PP) oil/water nanoemulsion, and AJ-NE was used for Jasmine oil blend (AJ) oil/water nanoemulsion.

### Characterization of nanoparticles and essential oils

#### X-rays diffraction

Powder X-ray diffraction (PXRD) patterns of LDH were investigated using X, Pert PRO Panalytical with Cu Kα radiation (λ = 1.5406 Å). Diffraction patterns were done at the 2θ range (4–80) with a scanning rate of 2.4°/min.

#### Nanoemulsion Droplet size and surface charge

Hydrodynamic radius and polydispersity index (PDI) were measured by dynamic light scattering (DLS) at an angle of 173° a room temperature of 25 °C. The surface charge or zeta potential investigated the frequency shift of scattered light at a scattering angle of 12°. Radius, PDI, and Zeta potential were analyzed using Zetasizer nano Zs analyzer (Malvern instruments) at the Egyptian Petroleum Research Institute (EPRI), Cairo, Egypt. Microemulsion (5 mg) was dispersed in 10 ml of distilled water at 25 °C.

#### Surface Morphology by Transmission Electron Microscope (TEM)

The internal morphology visualization of AJ and PP nanoemulsions was investigated using field transmission electron microscopy (HR-TEM, JSM-7100F) at EPRI where images were recorded with JEOL JEM-2100–115 high-resolution transmission electron microscopes with accelerating voltage 200 kV. 1 µL of nanoemulsion (n.e) diluted with double distilled water (1:200) and placed on a 200-mesh carbon-coated grid and left for 2 min. The excess liquid was adsorbed by cellulose filter paper. A drop of 2% (w/w) phosphotungstic acid (PTA) was added to the grid for 10 s to achieve negative staining; the excess PTA was disposed via adsorption on a filter paper.

#### Drug loading capacity (DL) and entrapment efficiency (EE) of EOs nanoformulation

The Entrapment efficiency (EE) of microemulsion encapsulated PP and AJ oils was estimated by measuring the amount of free un-encapsulated oil through UV-spectrophotometric^95–97^ technique at 237 and 260 nm (the maximum absorption peak of PP and AJ oils, respectively). EE is defined as the percentage of the drug that is successfully entrapped into the nanoparticles and calculated by the following equation:

$${\text{EE }} = \left( {{\text{Ct }} - {\text{ Cf}}} \right)/{\text{Ct}} \times { 1}00$$

$${\text{DL}} = \left( {{\text{Ct}} - {\text{Cf}}} \right)/{\text{wt}}$$

where Ct is the total amount of oil and Cf is the amount of free oil. The amount of free oil was determined by collecting microemulsion via centrifugation ultrafiltration^96,97^. One milliliter of freshly prepared oil microemulsion was diluted ten times (10 ml) with deionized water and 2 ml of each diluted sample was placed in a centrifuge tube (Amicon Ultra 5000 MWCO, Millipore, USA) and then centrifuged at 9000 rpm for 30 min. Using a super cooling centrifuge (Neofuge 15 heal force RTS laboratory high-speed centrifuge, Shanghai, China). As some of the free oil may be adsorbed to the membrane of the ultrafiltration tube to a certain extent^98^, the adsorption ability of the PP and AJ raw oils to the ultrafiltration membrane was investigated by the filtration of oil solution, known concentrations through the membrane, and measuring the concentration before and after filtration. The oil adsorbed fraction was anticipated to make free oil evaluation errors, due to adsorption, diminished. Free oil (in one milliliter nanoemulsion) included in the filtrate was measured spectrophotometrically at *λ*_max_ = 237 and 260 nm for PP and AJ, respectively, using a Jenway 635,001 6305 UV/Visible Spectrophotometer. The amount of the entrapped oil in one- milliliter oil nanoemulsion dispersion was determined by subtracting the amount of free drug (for one milliliter of oil nanoemulsion before dilution) from the total drug incorporated. The total amount of PP or AJ encapsulated oils in 1 ml nanoemulsion was investigated after the addition of 9.0 ml of methanol: chloroform 3:1 mixture to dissolve the oil-encapsulated nanoemulsion. The resultant solution was assessed for the total oil content spectrophotometrically using methanol as a blank at the same wavelengths.

#### Gas chromatography Mass spectroscopy (GC/MS)

The GC/MS analysis was performed by Thermo Scientific, Trace GC Ultra / ISQ Single Quadrupole MS, TG-5MS fused silica capillary column (30 m, 0.251 mm, 0.1 mm film thickness). An electron ionization system (ionization energy of 70 eV) was used for GC/MS detection. Helium was used as a carrier gas with a constant flow rate of 1 ml/min. The injector and MS transfer line temperature was set at 280 °C. The oven temperature was programmed initially as 50 °C (hold for 2 min) to 150 °C at an increasing rate of 7 °C /min; then to 270 at an increasing rate of 5 °C /min (hold for 2 min); and subsequently to 310 °C as a final temperature at an increasing rate of 3.5 °C /min (hold 10 min). The quantification of the identified components was investigated using a percent relative peak area. Tentative identification of the compounds was analyzed by comparing their relative retention time and mass spectra with those of the NIST, WILLY library data of the GC/MS system. Moreover, the identification was accomplished using computer search user-generated reference libraries, incorporating mass spectra. Peaks were examined by a single-ion chromatographic reconstruction to confirm their homogeneity. In some cases, when identical spectra had not been found, only the corresponding component's structural type was proposed based on its mass spectral fragmentation. Reference compounds were co-chromatographed, when possible, to confirm the GC retention times.

### Entomology

#### Culex pipiens colony

*Culex pipiens* larvae were obtained from a colony reared at the Medical and Molecular Entomology Section, Faculty of Science, Benha University, Egypt at 27 ± 2 °C, 70 ± 10% RH, and a 12:12 h (L/D) photoperiod and fed on fish food (Tetramin®) and powder bread daily (Baz, 2013).

#### Concentration- response bioassays

The essential oils and their nanoformulations were tested to evaluate their larvicidal efficacy against the second (L2) and fourth (L4) instar larvae of *Cx. pipiens*^61,62^. Oils were diluted in a solvent (5% Tween 80 diluted in water) to make 1% stock solution. Six concentrations were prepared for oils and nanoformulations (62.5, 125, 250, 500, 1000, and 2000 ppm). The control groups were treated with solvent; LDH-free and NE-free formulations. Twenty mosquito larvae were used for each replicate and three replicates (60 larvae) were used for each concentration. Temephos 50% (EC) was used as a positive control for comparison with three replications, it was manufactured by KZ Pesticides and Chemicals in Egypt**.** Larval mortalities (MOs) were recorded 24 h post-treatment (PT).

### Biochemical assay

#### Preparation of specimens

Biochemical assays after the application of the LC_50_ values, calculated from the previous larvicidal tests, were made to evaluate the effect of oils and their nanoemulsions against *Cx. pipiens* (L4), 24 h PT, regarding the levels of some enzymes such as phenolxidases and non-specific esterase. Ten larvae per replicate were homogenized (3 replicates were used for each test) in an ultrasonic homogenizer, and then the homogenates were centrifuged for 15 min in a cooling centrifuge (5 °C, 8000 rpm). The supernatants were frozen until used^99^**.**

#### Phenoloxidase activity

The activity of phenoloxidase was measured by using a mixture of 200 μl enzyme solution, 0.5 ml phosphate buffer (0.1 M, pH 7), and 200 μl catechol solution (2%). At 25 °C, the substrate was incubated. The enzyme reaction was started by adding a catechol solution. After exactly 1 min, the optical density was measured. Zero adjustment was made against the sample blank at 405 nm^100^.

#### Non-specific esterase

The activity of Alpha esterases (α-esterases) and beta esterases (β-esterases) were determined by using α- naphthyl acetate or β-naphthyl acetate as substrates, respectively. The procedure color was red at 600 or 555 nm for α- and β-naphthol produced from hydrolysis of the substrate, respectively^101^.

### Data analysis

Data were analyzed through Two-Way Analysis of Variance (ANOVA), Duncan's multiple range tests, and Probit analysis for calculating the lethal concentration values using the computer program PASW Statistics 2009 (SPSS version 22). The relative toxicities were calculated^42^.

$${\text{Relative toxicity}} = {\text{ LC}}_{{{5}0}} {\text{of the least toxic material}}/{\text{ LC}}_{{{5}0}} {\text{of each tested material}}.$$

## Results and discussion

### Synthesis of Hierarchical 3D CoLDH and ZnAl LDH

#### XRD

**Figure 1:**
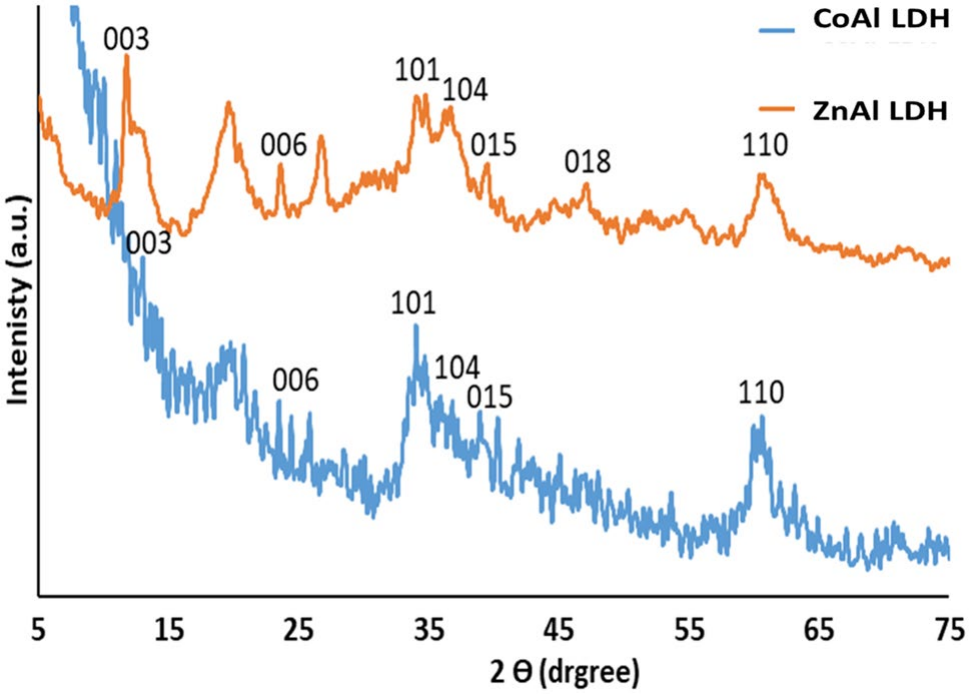
XRD pattern of synthesized Co LDH and Zn LDH.

The crystal structure of the synthesized CoAl LDH and ZnAl LDH were identified by XRD (Fig. 1). The XRD of both CoAl LDH and ZnAl-LDH exhibited a Rhombohedral crystal structure with seven diffraction peaks located at 2 theta of 11.73°, 23.58°, 33.97°, 37.39°, 39.31°, 46.91°, and 60.33° that corresponding to various diffraction planes (003), (006), (101), (104), (015), (018), and (110), respectively. The positions of diffraction peaks were consistent with that of the ICDD (The International Centre for Diffraction Data) reference card 04-014-8855 for cobalt, aluminum carbonate hydroxide hydrate, and 00-063-0586 for zinc aluminum carbonate hydroxide hydrate of hydrotalcite family, indicating the successful preparation of both CoAl LDH and ZnAl LDH.

#### Detection of released active ingredients from LDH-loaded E.O (Drug release)

**Figure 2:**
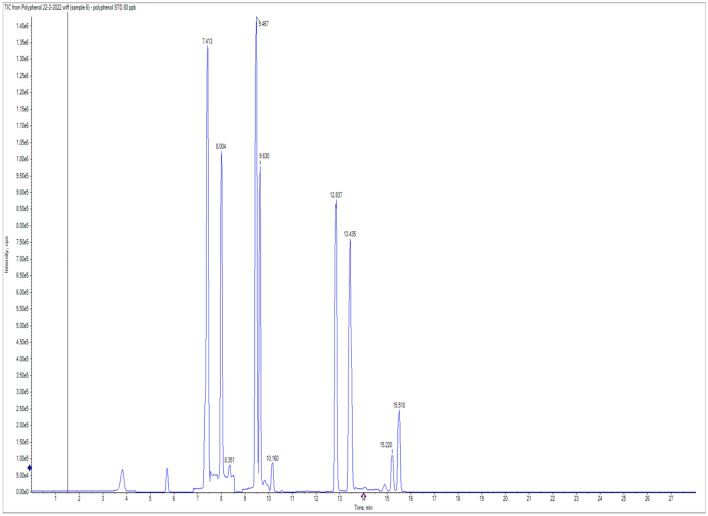
LC/MS/MS Chromatogram of standards.

**Figure 3:**
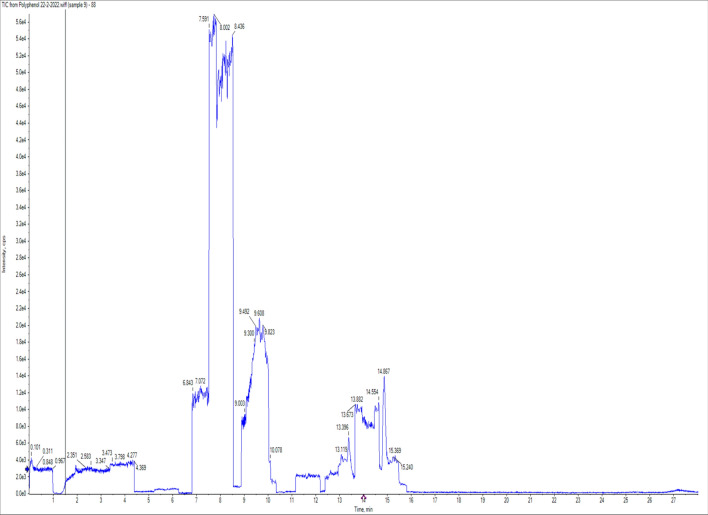
LC/MS/MS Chromatogram of CoAl LDH loaded MA oil.

**Figure 4:**
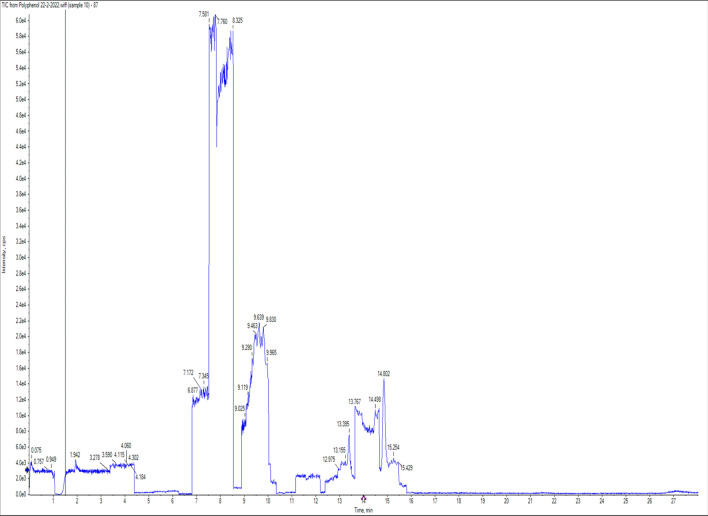
LC/MS/MS Chromatogram of ZnAl LDH loaded peppermint oil.

To identify the active ingredients released from Co and ZnAl LDHs loaded by PP and AJ essential oils, this study was performed on CoAl LDH and ZnAl LDH loaded peppermint essential oils as CoAl LDH (PP) and ZnAl LDH (PP). Absolute ethanol, as a polar solvent, was used to trap the nonvolatile polar compounds and normal hexane was used to detect the volatile non-polar compounds if released or even present. The solid slurry of LDH collected by centrifugation and supernatant in each case was concentrated and analyzed using GC/MS to determine nonpolar volatile active ingredients. The other portion was injected directly into LC–MS/MS, to determine the polar nonvolatile active ingredients. For the volatile nonpolar active ingredients, the GC/MS analysis showed humps related to the device background, and no significant compounds were detected. Such outcomes could be due to the instability of the nonpolar volatile active ingredients as they vaporized quickly, and/or the chances of loading were very low and being chargeless components that may introduce a convenient interpretation of why such active ingredients did not exist in the GC/MS Chromatogram. For the polyphenols and flavonoids, polar nonvolatile active ingredients, the results of LC MS/MS showed the existence of few active ingredients such as naringenin (46.7 ng/ml); ellagic acid (10.87 ng/ml); and Luteolin (0.44 ng/ml) (Table 1) and (Figs. 2 and 3) for Co LDH loaded peppermint oil and naringenin (46.9 ng/ml); ellagic acid (11.57 ng/ml); and Luteolin (0.48 ng/ml) for Zn LDH loaded peppermint oil (Table 2) and (Figs. 2 and 4).

**Table 1:** CoAl LDH (PP) polyphenols and flavonoids drug release detection.

Compound	Standard			CoAl LDH (PP)		
	STD Area	RT	ng/ml	Area	RT	ng/ml
Chlorogenic acid 355.1/163	668,900	7.31	80	ND	ND	ND
Daidzein 255.1/199	3,142,000	12.84	80	ND	ND	ND
Gallic acid 168.9/124.9	446,000	3.83	80	ND	ND	ND
Caffeic acid 178/135	4,999,000	8.02	80	ND	ND	ND
Rutin 609/299.9	2,810,000	9.65	80	ND	ND	ND
Coumaric acid 162.9/119	7,477,000	9.48	80	ND	ND	ND
Vanillin 151/136	115,400	9.5	80	ND	ND	ND
Naringenin 271/119	61,880	14.91	80	36,330	14.88	46.96833
Querectin 301/151	2,015,000	13.49	80	ND	ND	ND
Ellagic acid 301/145	47,300	9.86	80	6841	9.85	11.57040
3.4-Dihydroxybenzoic acid 152.9/109	382,600	5.72	80	ND	ND	ND
Hesperetin 301/136	1,007,000	15.52	80	ND	ND	ND
Myricetin 317/137	5011	11.64	80	ND	ND	ND
Cinnamic acid 146.9/102.6	44,290	14.09	80	ND	ND	ND
Methyl gallate 183/124	6,739,000	7.42	80	ND	ND	ND
Kaempferol 284.7/93	416,600	15.24	80	ND	ND	ND
Ferulic acid 192.8/133.9	299,700	10.18	80	ND	ND	ND
Syringic acid 196.8/181.9	99,270	8.36	80	ND	ND	ND
Apigenin 269/151	23,740	14.95	80	ND	ND	ND
Catechin 288.8/244.9	183,600	7.32	80	ND	ND	ND
Luteolin 284.7/132.9	3,174,000	13.42	80	19,260	13.43	0.48544

**Table 2:** ZnAl LDH (PP) polyphenols and flavonoids drug release detection.

Compound	Standard			ZnAl LDH (PP)		
	STD Area	RT	ng/ml	Area	RT	ng/ml
Chlorogenic acid 355.1/163	668,900	7.31	80	ND	ND	ND
Daidzein 255.1/199	3,142,000	12.84	80	ND	ND	ND
Gallic acid 168.9/124.9	446,000	3.83	80	ND	ND	ND
Caffeic acid 178/135	4,999,000	8.02	80	ND	ND	ND
Rutin 609/299.9	2,810,000	9.65	80	ND	ND	ND
Coumaric acid 162.9/119	7,477,000	9.48	80	ND	ND	ND
Vanillin 151/136	115,400	9.5	80	ND	ND	ND
Naringenin 271/119	61,880	14.91	80	36,110	14.87	46.68390
Querectin 301/151	2,015,000	13.49	80	ND	ND	ND
Ellagic acid 301/145	47,300	9.86	80	6428	9.84	10.87188
3.4-Dihydroxybenzoic acid 152.9/109	382,600	5.72	80	ND	ND	ND
Hesperetin 301/136	1,007,000	15.52	80	ND	ND	ND
Myricetin 317/137	5011	11.64	80	ND	ND	ND
Cinnamic acid 146.9/102.6	44,290	14.09	80	ND	ND	ND
Methyl gallate 183/124	6,739,000	7.42	80	ND	ND	ND
Kaempferol 284.7/93	416,600	15.24	80	ND	ND	ND
Ferulic acid 192.8/133.9	299,700	10.18	80	ND	ND	ND
Syringic acid 196.8/181.9	99,270	8.36	80	ND	ND	ND
Apigenin 269/151	23,740	14.95	80	ND	ND	ND
Catechin 288.8/244.9	183,600	7.32	80	ND	ND	ND
Luteolin 284.7/132.9	3,174,000	13.42	80	17,510	13.42	0.44134

#### SEM Morphology of LDH and LDH loaded Essential oils.

**Figure 5:**
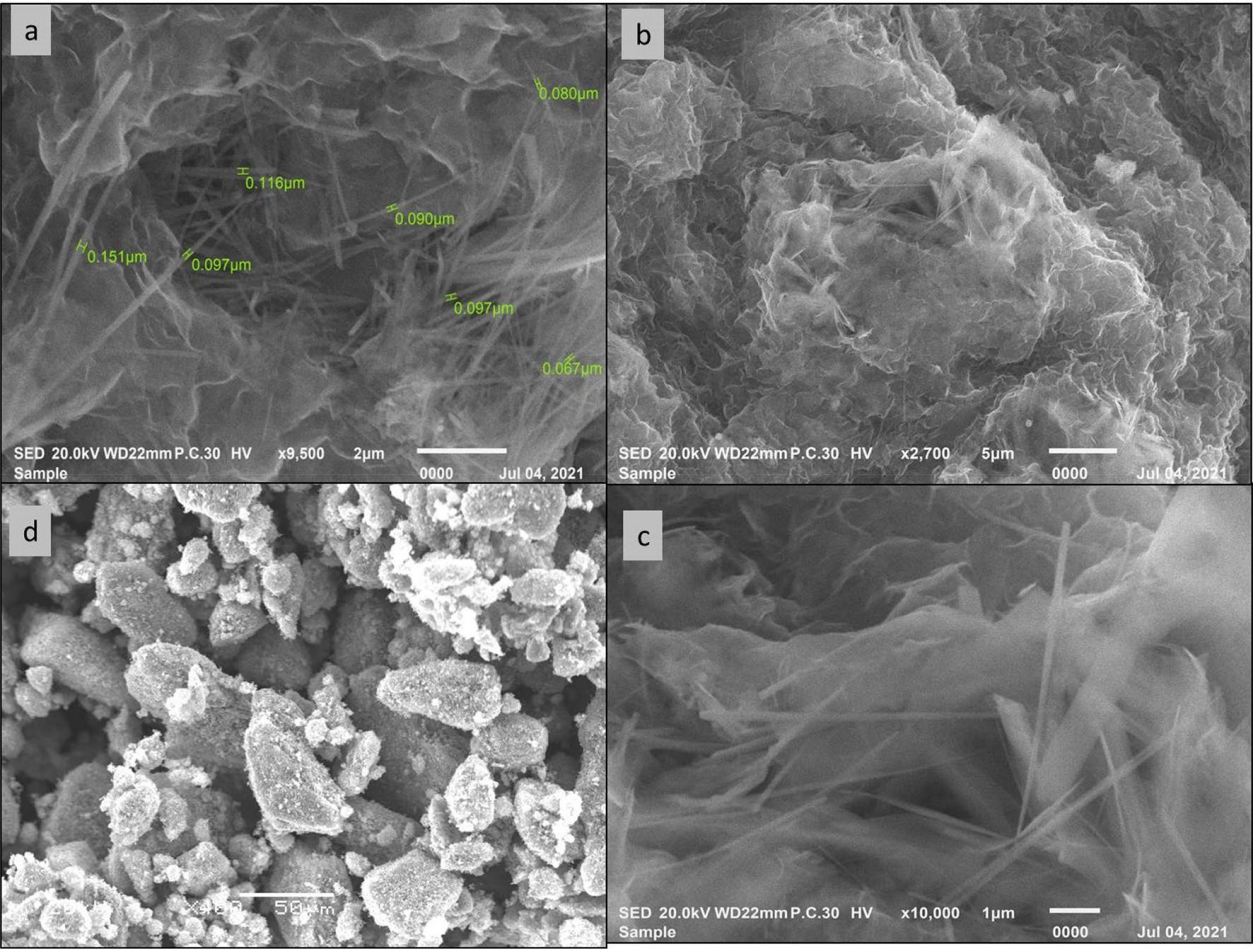
SEM surface topography of flower shape 3-D CoAl LDH (**a**, **b** & **c**) and ZnAl LDH (**d**) before loading.

**Figure 6:**
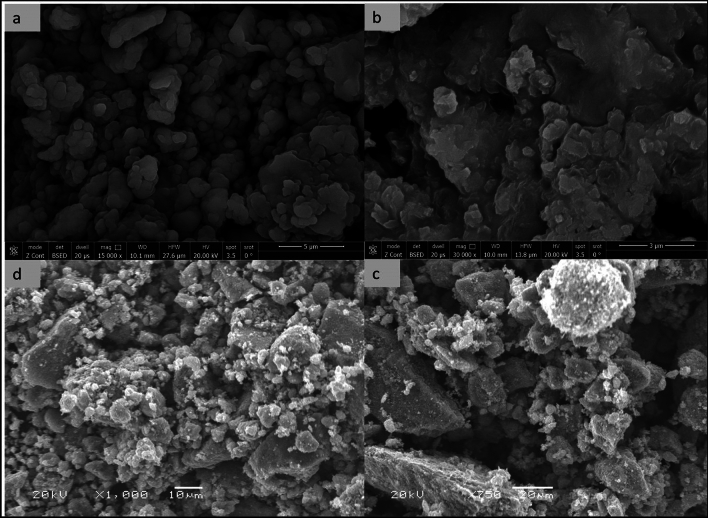
SEM surface topography of Co&Zn LDH loaded with different oils; CoAl LDH (PP) (**a**), CoAl LDH (AJ) (**b**), ZnAl LDH (AJ) (**c**), and ZnAl LDH (PP) (**d**).

The morphological and microstructural investigations of LDHs were done using scanning Electron Microscopy (SEM). The morphology of the synthesized CoAl LDH depicted a well-designed three-dimensional flower-like nanostructure composed of many ultra-thin nanosheets with border size dimensions varied from 60 to 120 nm (Fig. 5a,b). The construction of such nanoparticles (Fig. 5c), attributed to the CO_2_ gas released during the hydrolysis of urea within the synthesis process, had its impact, on improving the gas sensing via enhancing surface area^102^. While ZnAl LDH showed a plate-like shape with little bulky segments (Fig. 5d). After loading of the essential oils, LDHs showed more bulky aggregations (Fig. 6b–d) and the flower shape was slightly reserved (Fig. 6a). Such relative size enlargement may be owing to the high aggregations and not necessarily came from loading of more active ingredient. Moreover, the drug release study evaluated the released active ingredients concentration by the LC/MS/MS technique and a modest concentration of polyphenols and flavonoids have been found. The reason for that may be related to the nature of the active ingredient, meaning that, the LDH as a carrier is not compatible with the active ingredient in PP as the LDH nanocarriers were preferably used for gas sensing like CO_2_ and NO_2_^103^, and the loading of negatively charged molecules like dyes, pigments^104^, nucleic acid^105^ and some enriched essential oil with polyphenols and flavonoids like water-soluble green tea oil^61^.

### Synthesis of Oil / Water Nanoemulsion

#### Droplet size (DLS) and zeta potential (Z.P)

**Figure 7:**
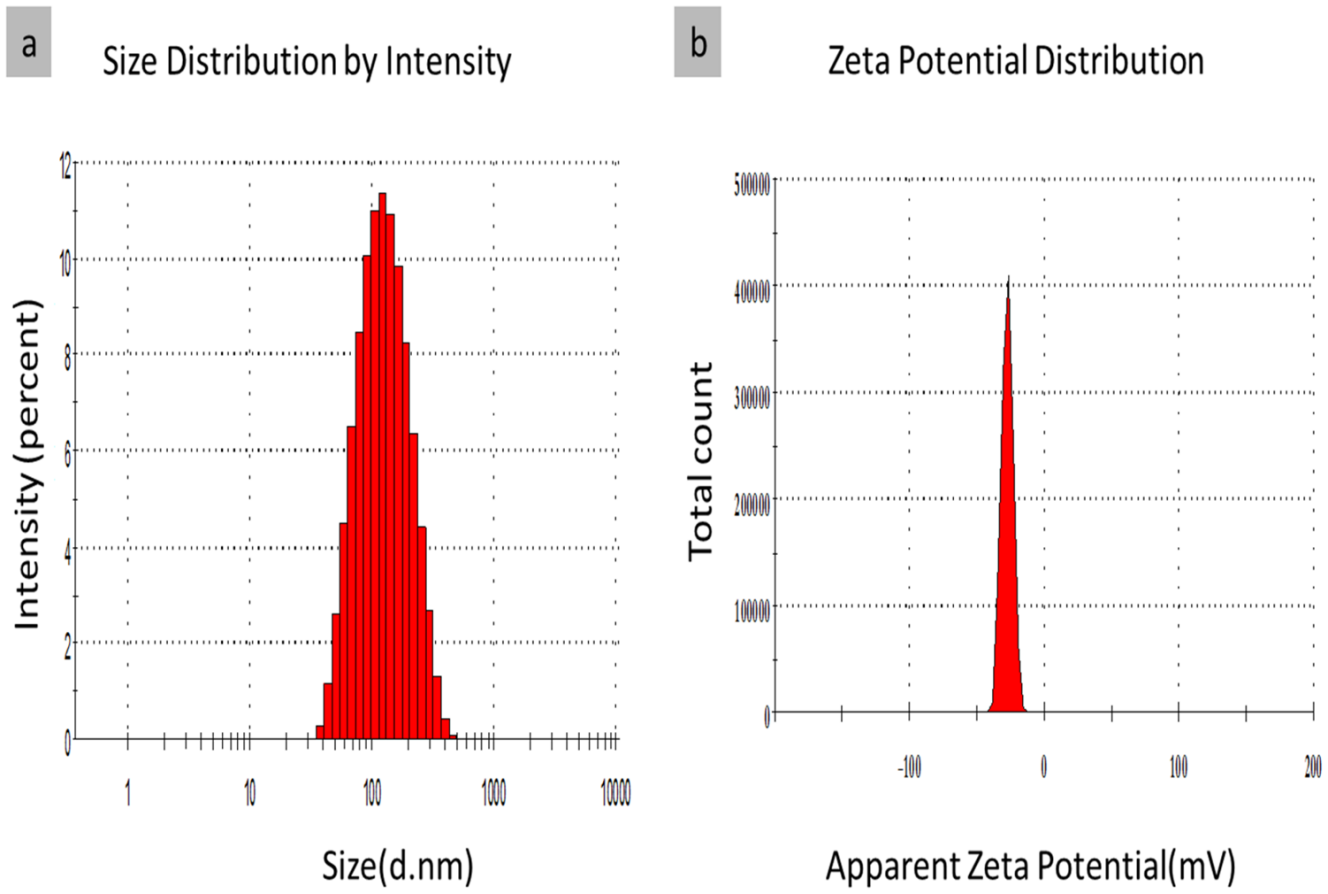
Droplet size and Zeta potential of jasmine (AJ) oil nanoemulsion (AJ-NE).

**Figure 8:**
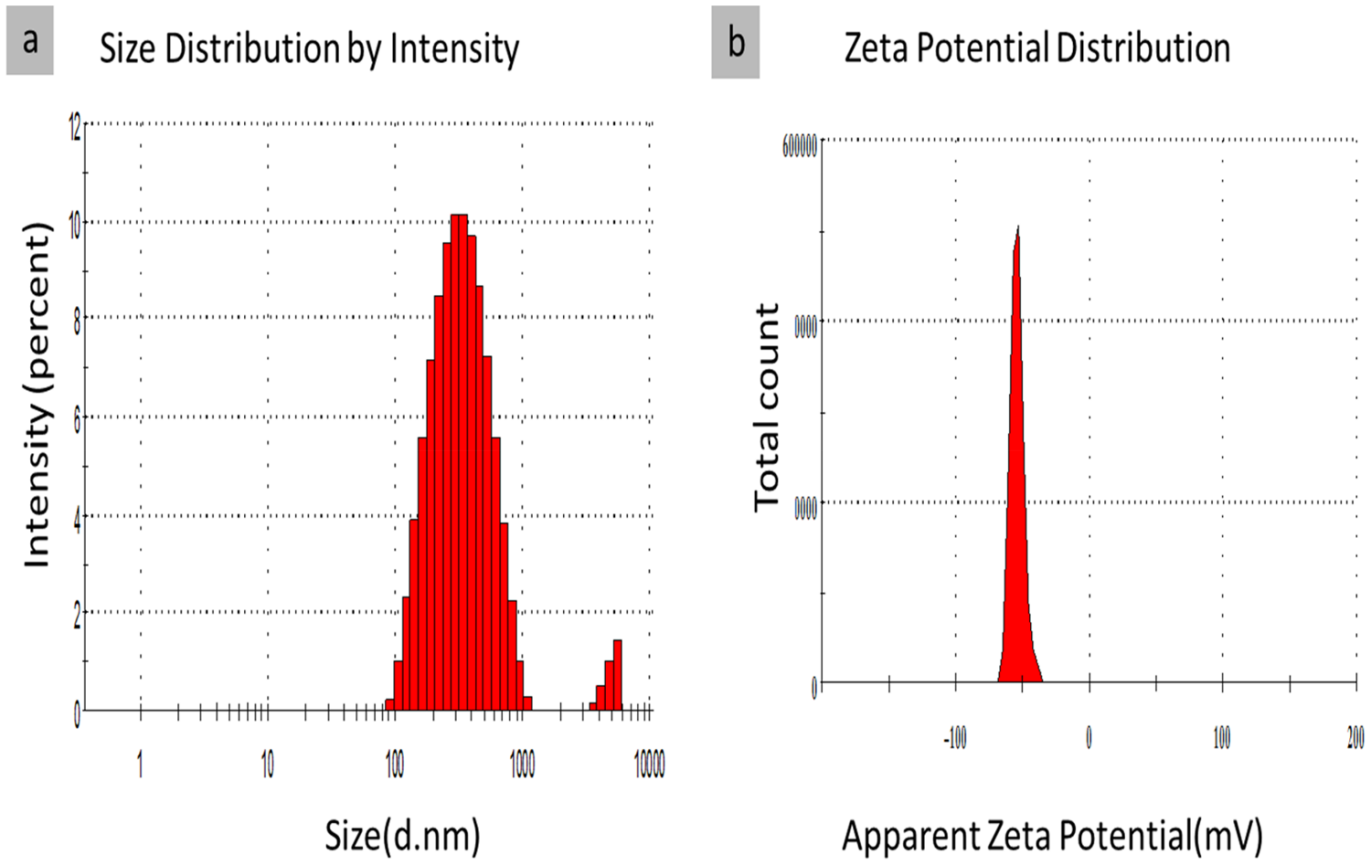
Droplet size and Zeta potential of peppermint (PP) oil nanoemulsion (PP-NE).

Dynamic Light Scattering evaluated the z-average mean diameter of the nanoparticles^106^. The average particle size and Poly Dispersity Index of AJ-NE and PP-NE oil nanoemulsions were described (Figs. 7 and 8). It was observed that nanoemulsion-encapsulated AJ oil showed relative smallest average particle size (199 nm), whereas the nanoemulsion-encapsulated PP oil displayed a mean particle size of 333 nm. Poly Dispersity Index (PDI) exhibited whether the nanoparticles were homogenous or heterogeneous and measured the ratio mass average of the molecular mass to the average molecular mass, a range from 0 for perfectly uniform samples with no size variations (monomodal or monodispersed) to 1 for highly varied particle size. As this ratio became smaller, the solution became more homogenous. A small numerical value of PDIs indicated a narrow size distribution (Table 3)^107^, while a higher value of 0.5 indicated, a more broadened size distribution^108^. All of the prepared AJ and PP nanoemulsions in this study showed low PDI values (less than 0.3) 0.249 and 0.198, respectively.

**Table 3:** DLS and Zeta of oil-loaded nanoemulsion.

Microemulsion	DLS/ nm	Zeta/ mV	PDI
AJ -NE	199.2	-27.4	0.249
PP-NE	333.8	-54.1	0.198

AJ (Jasmine oil blend); PP (Peppermint oil).

Nanoemulsion charge and stability were measured by measuring the change in the net charge (zeta potential), where the best values of Z.P are >  + 30 and <  − 30. Moreover, AJ and PP nanoemulsions, depicted Z.P values of − 27 mV and − 54 mV, respectively. Furthermore, AJ-NE reflected good stability, while PP-NE nanoemulsion presented a unique stability behavior due to the increase of the negative charges making extensive repulsion forces between particles and that will diminish the aggregation ability. In general, when the number of charges decreased, the value of zeta potential became less positive than + 30 or less negative than − 30, and the particles tends to agglomerate or aggregates making DLS and PDI values increases and the particles become larger and slower in their mobility and the system became more heterogeneous. Elevated values of Z.P in addition to small values of DLS and PDI, especially for PP nanoemulsion, confirmed the stability of nanoemulsion prepared as a homogenous system and it was consistent with the upcoming results of TEM.

#### Internal morphology by TEM

**Figure 9:**
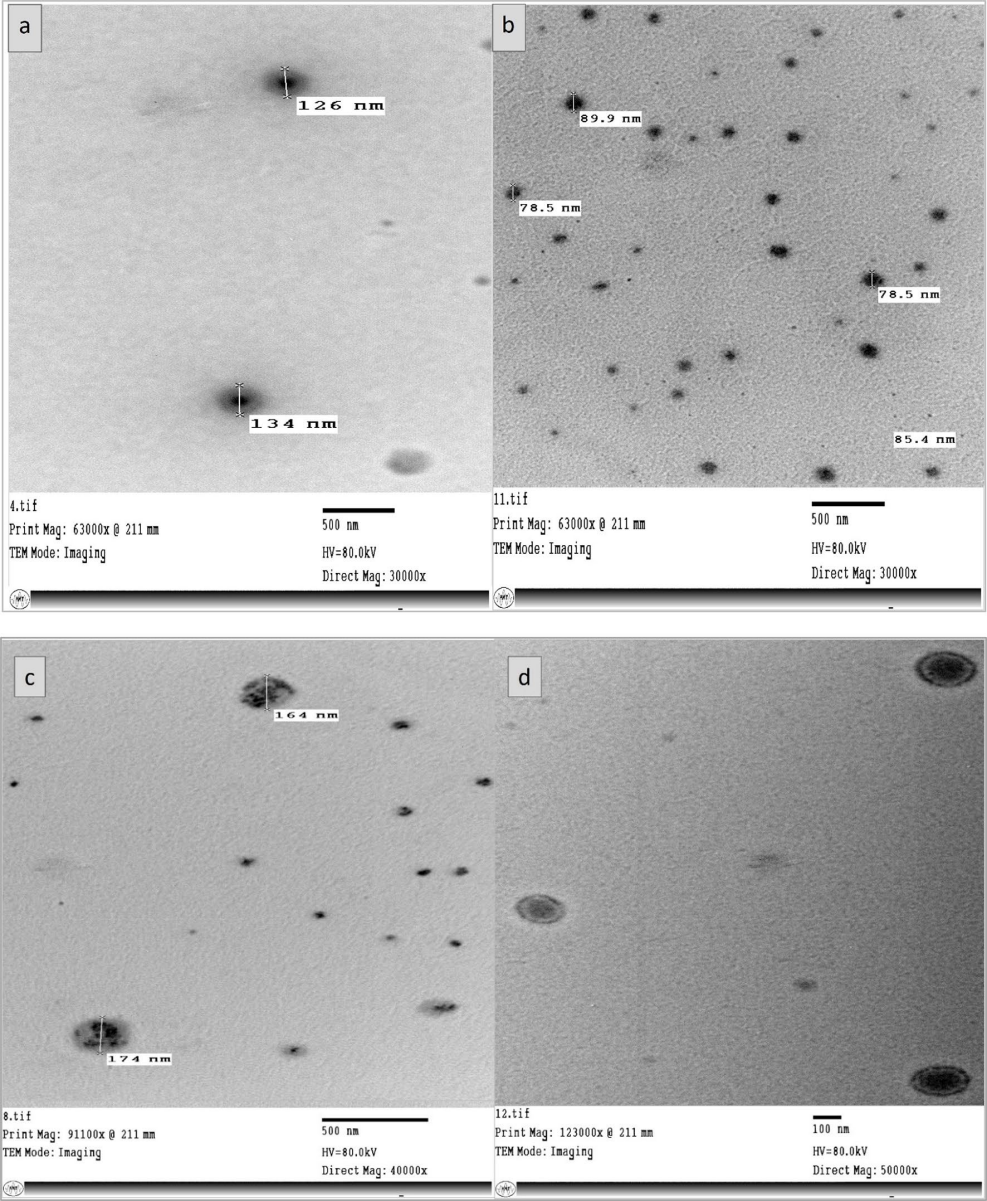
(**A**) TEM morphology of Arabian jasmine essential oil nanoemulsions: (**a**, **b**) AJ-NE. (**B**). TEM morphology of peppermint essential oil nanoemulsions: (**c**, **d**) PP-NE.

Transmission Electron microscopy is one of the most effective ways to describe the details of internal structure, size distribution, and particle morphology of nanoparticles^109,110^. TEM imaging elucidated the morphology of AJ and PP nanoemulsions, which presented uniform, smooth, and spherical shapes with vesicular sizes in the range of 70–180 nm without observable aggregations and comparatively some other smaller and spherical droplets, their sizes extended to 20 nm, were existed. Such results was found to be consistent with the values of Z.P and PDI (Fig. 9a–c). The encapsulated vesicle of PP oil with droplet size in the range of 200 nm was observed (Fig. 9d), where the inner layer represented the oil droplet, and the outer layer represented the surfactant monolayer. The paradox of some droplets' size described by TEM is not confused with the particle size presented by DLS. TEM imaging measured each particle separately, while the particle size by DLS was calculated based on the average ratio. In other words, DLS measured the average size of particles without assessing a specific particle size. The encapsulation process appeared clearly in Fig. 9A,B indicating successful loading of AJ and PP oils, respectively as hydrophobic phase in the inner layer surrounded by water and surfactant hydrophilic components in the outer shell layer to form oil in water nanoemulsion.

#### Drug loading capacity and entrapment efficiency

The entrapment efficiency (EE) or the percentage of drug content was successfully entrapped into the nanoparticles and calculated as the ratio between the actual quantities of drug entrapped divided by the total drug added. On the other hand, the drug loading capacity (DLC) was defined as the amount of drug loaded per unit weight of the nanoparticle, indicating the percentage of the mass of nanoparticle that is due to the encapsulated drug and calculated by the amount of total entrapped drug divided by the total nanoparticle weight.

The Encapsulation Efficiency percentage (%) and drug loading capacity of AJ and PP oil in nanoemulsion were determined in this study after separation of the free oil (unloaded) from the nanoemulsion suspension by ultrafiltration centrifugation^98^. The average entrapment efficiencies were found to be 76.9 ± 2.3 and 83.2 ± 2.4%, and drug loading capacities were 17.5 ± 0.49 and 18.13 ± 1.01% for AJ-NE and PP-NE, respectively (Table 4). As a result, most of the oil was entrapped in the nanoemulsion system. Such high entrapment efficiencies might be attributed to the extensive hydrophobic characteristics of the encapsulated oils, which are associated with liquid lipid core^111^. Because of the hydrophobic nature of AJ and PP oils, their higher affinity and solubility in oleic acid, such oils were expected to be entrapped within the oil channel of nanoemulsion. Consequently, the delayed leakage of the loaded oils, from the oil channels in the nanoemulsion droplet, to the surrounding aqueous phase, through the preparation and centrifugation processes^112^, may introduce a convenient interpretation of the higher entrapment efficiency percentage obtained by AJ-NE and PP-NE oils.

**Table 4:** The encapsulation efficiency and loading capacity of the prepared nanoemulsion.

Nano Sys	Encapsulation Efficiency (%)					
	Replicate.1	Replicate.2	Replicate.1	Aver	St. dev	St. Error
AJ-NE	73.6	81.4	75.8	76.93333	4.021608	2.321877
PP-NE	79.5	87.9	82.3	83.23333	4.277071	2.469368
Drug Loading Capacity (%)						
AJ-NE	16.9	17.2	18.5	17.53333	0.85049	0.491031
PP-NE	18.3	19.8	16.3	18.13333	1.755942	1.013794

#### Phyto-chemical analysis of Peppermint and Jasmine oil blend

The chemical constituents of AJ and PP were identified by GC–MS analyses. Most compounds belonged to terpenes and phenols in both oils. The oil blend of AJ contained 19 main chemical compounds, mainly linalyl acetate; (6.50), 4-aminohydrazide benzoic acid (3.19); and Àazidoàmethylcyclpent aneacetaldehyde (2.38) (Table 5). Meanwhile, GC–MS analyses showed that PP oil had more than 25 main chemical compounds, mostly 1-Methylethylcyclopentane (26.96); ( +)-isomentol (13.41); 4-Oxatricyclo [5.2.1.0 (2,6)] decan-8-ol (13.21); and 2-α-Pinene (4.73) (Table 6).

**Table 5:** GC–MS analysis of the Jasmine oil blend.

No	Rt (min.)	MW	MF	Area %	Probabilities of the detected compounds
1	9.17	136	C_10_H_16_	1.11	3,4-Dimethyl-1,5-cyc looctadiene
2	11.34	196	C_12_H_20_O_2_	6.50	linalyl acetate
3	11.85	230	C_15_H_18_O_2_	0.85	1-Benzyloxymeth, yl1hydroxymethyl-2,5-cyclohexadiene
4	15.74	167	C_8_H_13_N_3_O	2.38	Àazidoàmethylcyclpent aneacetaldehyde
5	17.45	180	C_13_H_24_	0.75	2-methyl, trans 1,1'-Bicyclohexyl,
6	17.73	222	C_16_H_30_	0.73	8-Hexadecyne
7	18.09	127	C_7_H_13_NO	1.25	3-methyl hexahydro *2H*Azepin2one
8	18.16	151	C_7_H_9_N_3_O	3.19	4-aminohydrazide Benzoic acid
9	18.42	240	C_17_H_36_	1.18	2,6,10-trimethyl Tetradecane,
10	18.66	194	C_14_H_26_	1.06	PMenthane,3-(2-methylpropen-1-yl)
11	19.12	232	C_10_H_20_N_2_S_2_	0.75	1,1'-dithiobis Piperidine
12	19.53	194	C_14_H_26_	1.10	4,4'-dimethyl-1,1'-Bicyclohexyl
13	20.05	306	C_20_H_34_O_2_	0.74	4,14-Didecarboxy-l4-methyl-13,14-di(hydroxymethyl)isoagathic acid
14	20.19	282	C_18_H_34_O_2_	0.84	*E*10-Methyl-11-tetradec En-1-ol propionate
15	20.60	208	C_11_H_12_O_2_S	1.54	4(Ethylthio)-6,7-dihydroBenzofuran-5-carboxaldehyde
16	20.70	240	C_17_H_36_	0.95	2,6,10-trimethyl Tetradecane
17	20.94	198	C_12_H_22_O_2_	1.27	6-heptyltetrahydro 2HPyran-2-one
18	21.12	254	C_18_H_38_	0.92	2,2,4,9,11,11-hexamethyl Dodecane
19	21.74	236	C_15_H_29_BO	0.84	*(Z)-*13-propox -13-Borabicyclo[7.3.0]tridecane

**Table 6:** GC–MS analysis of Peppermint oil.

No	Rt (min.)	MW	MF	Area %	Probabilities of the detected compounds
1	5.17	170	C_10_H_18_O_2_	0.37	N-HEXYL-CIS-CROTONATE
2	6.37	136	C_10_H_16_	1.28	α-Thujene
3	6.46	136	C_10_H_16_	3.92	α-Pinene
4	7.64	136	C_10_H_16_	4.73	2-α-Pinene
5	8.13	196	C_12_H_20_O_2_	0.62	Linalyl acetate
6	9.2	154	C_9_H_14_O_2_	13.22	4-Oxatricyclo[5.2.1.0(2,6)]decan-8-ol
7	12.87	112	C_8_H_16_	26.96	1-methylethyl Cyclopentane
8	13.31	154	C_10_H_18_O	3.95	l-Menthone
9	13.66	156	C_9_H_16_O_2_	1.80	Bicyclo[3.3.1]nonane-2,6-diol
10	14.27	156	C_10_H_20_O	13.41	( +)-Isomenthol
11	15.75	150	C_10_H_14_O	2.06	2-Cyclohexen-1-one,2-methyl-5-(1-methylethenyl) (CAS)
12	16.87	228	C_13_H_24_O_3_	2.00	3,7-Dimethyloct-6-enylethyl carbonate
13	19.97	204	C_15_H_24_	1.03	Trans-Caryophyllene
14	21.97	212	C_15_H_32_	0.43	Pentadecane
15	22.90	155	C_9_H_17_NO	1.40	1-isopropyl-5-methyl Azacyclohexan-3-one
16	23.01	248	C_12_H_25_Br	0.64	2-Bromo dodecane
17	23.43	268	C_19_H_40_	0.83	2,6-dimethyl Heptadecane
18	23.87	310	C_22_H_46_	0.45	Docosane (CAS)
19	24.10	226	C_16_H_34_	0.96	Hexadecane (CAS)
20	24.29	254	C_18_H_38_	1.20	8-methyl Heptadecane (CAS)
21	25.14	298	C_20_H_42_O	1.66	Diisodecyl ether
22	25.71	362	C_20_H_42_O_3_S	1.20	Sulfurous acid, pentadecyl pentyl ester
23	26.34	212	C_15_H_32_	0.49	Pentadecane
24	26.50	173	C_10_H_23_NO	1.16	O-decyl hydroxylamine
25	27.30	250	C_17_H_30_O	1.83	Podocarpan-α-ol

It is well known that plant essential oils have varying toxicity to insects and other pests because they contain many active secondary compounds that distinguish each type from others. It was revealed that sesquiterpenes, fatty acid esters, and phenols were the most prevalent components in essential oils. Parallel to our findings, phytochemicals as flavonoids, alkaloids, esters, glycosides, and fatty acids could play rules against insects (as toxicants, attractants growth retardants, repellents, chemosterilants, and feeding deterrents/antifeedants^23,43,56,60,113,114^**.** The larvicidal effectiveness of *Allium sativum* oil against *Cx. pipiens* and *Culex restuans* were recorded (EC_50_ values of 2.7 and 7.5 g/ml, respectively). Allyl disulfide and diallyl trisulfide were found to be important components in the oil with 49.13 and 31.08% relative proportions, respectively^115^.

The use of phytochemicals can improve the effectiveness of biological control agents. These plant compounds could help in the development of more effective insect pest control agents because they are less expensive, easily biodegradable, and are considered highly suitable for integrated pest management programs being active against a variety of insect pests. Because it has biologically active secondary chemicals such as terpenes, tannins, fatty acid esters, phenols, and other compounds that affect insect pests in a distinct way^43,60,116^. These phytochemical compounds such as alkaloids and phenolics are associated with toxicity because they are essential in the interactions between plants and herbivores and pathogens, and their antioxidant qualities^43,60,116^ are thought to be the trigger of the pesticide effect^117^.

### Larvicidal bioassay

#### Concentration response bioassays

Using botanicals, including essential oils as larvicides is a promising field of research^23,49,50,118^. In this study, the larvicidal effects of PP and AJ and their nanoformulations were evaluated against the 2nd and early 4th larvae, suggesting an insecticidal activity against *Cx. pipiens*. Such results showed that the highest larval mortalities were observed PT with nanoemulsions rather than their corresponding oils, whereas low to good results were furnished by LDH-loaded oils. This study specified that the complete (100 MO%) larvicidal effects of AJ and PP oils and their Nano formulations were recorded PT of L2 and L4, 24 h PT at the highest concentration of 2000 ppm. On the other hand, MO% PT of L2 with 1000 ppm of AJ, Co LDH (AJ), Zn LDH (AJ), and AJ-NE were 91, 95, 93, and 100%, respectively, and the corresponding values of L4 were 83, 89, 86, and 100%, respectively. Meanwhile, MO% of L2 following treatment with PP, Co LDH (PP), Zn LDH (PP), and PP-NE were 94, 95, 95, and 100%, respectively, and those of L4 were 85, 91, 89, and 100%, respectively (Table 7). Also, free LDH caused 22.00 and 19 MO% of the treated 2nd and 4th instar larvae, respectively. In the same trend, the corresponding values for free NE were 18 and 15%, respectively. The negative control (99 ml of distilled water with 1 ml of Tween 80) was not toxic to all larvae, while the positive control (temephos) had significant effects on the mosquito larvae.

**Table 7:** The larvicidal effects of Jasmine oil blend and Peppermint oils and their Nano against *Culex pipiens,* 24 h post-treatment.

Oil name	Nano- Formulations	Stage	Mortality % (Mean ± SE)						
			Control	62.5*	125	250	500	1000	2000
Jasmine oil blend	Oil (AJ)	2^nd^	00 ± 0.0^aG^	8.0 ± 2.55^aF^	18.0 ± 2.55^aE^	38.0 ± 3.00^aD^	71.0 ± 4.30^aC^	91.0 ± 3.32^aB^	100 ± 0.00^aA^
		4^th^	00 ± 0.0^aG^	4.0 ± 1.87^bF^	10.0 ± 1.58^bE^	29.0 ± 2.92^bD^	61.0 ± 4.30^bC^	83.0 ± 2.55^bB^	100 ± 0.00^aA^
	Co LDH (AJ)	2^nd^	1.0 ± 1.0^aG^	9.0 ± 1.87^aF^	21.0 ± 1.87^aE^	42.0 ± 2.00^aD^	75.0 ± 2.74^aC^	95.0 ± 2.24^aB^	100 ± 0.00^aA^
		4^th^	1.0 ± 1.0^aG^	6.0 ± 1.00^bF^	15.0 ± 2.24^bE^	34.0 ± 2.92^bD^	66.0 ± 4.00^bC^	89.0 ± 2.45^bB^	100 ± 0.00^aA^
	Zn LDH (AJ)	2^nd^	2.0 ± 1.2^aG^	8.0 ± 2.00^aF^	20.0 ± 1.58^aE^	47.0 ± 3.00^aD^	75.0 ± 2.74^aC^	93.0 ± 2.00^aB^	100 ± 0.00^aA^
		4^th^	2.0 ± 1.2^aG^	5.0 ± 1.58^bF^	13.0 ± 3.39^bE^	32.0 ± 2.55^bD^	64.0 ± 4.00^bC^	86.0 ± 2.92^bB^	100 ± 0.00^aA^
	AJ -NE	2^nd^	2.0 ± 1.2^aF^	22.0 ± 2.00^aE^	45.0 ± 5.00^aD^	72.0 ± 6.44^aC^	96.0 ± 2.45^aB^	100 ± 0.00^aA^	100 ± 0.00^aA^
		4^th^	1.0 ± 1.0^bF^	16.0 ± 1.87^bE^	32.0 ± 2.55^bD^	55.0 ± 5.70^bC^	81.0 ± 3.67^bB^	100 ± 0.00^aA^	100 ± 0.00^aA^
	Free-LDH	2^nd^	00 ± 0.0^aG^	4.0 ± 1.87^aF^	8.0 ± 1.22^aE^	13.0 ± 1.22^aD^	18.0 ± 2.55^aC^	22.0 ± 3.00^aB^	31.0 ± 1.87^aA^
		4^th^	00 ± 0.0^aG^	1.0 ± 1.00^bF^	5.0 ± 2.24^bE^	10.0 ± 2.24^bD^	15.0 ± 3.16^bC^	19.0 ± 2.92^bB^	24.0 ± 2.45^bA^
	Free-NE	2^nd^	00 ± 0.0^aG^	3.0 ± 1.22^aF^	6.0 ± 1.87^aE^	10.0 ± 1.58^aD^	13.0 ± 2.00^aC^	18.0 ± 3.00^aB^	25.0 ± 2.74^aA^
		4^th^	00 ± 0.0^aG^	1.0 ± 1.00^bF^	4.0 ± 2.45^bE^	7.0 ± 2.55^bD^	10.0 ± 3.16^bC^	15.0 ± 2.74^bB^	20.0 ± 2.74^bA^
Peppermint	Oil (PP)	2^nd^	00 ± 0.0^aG^	9.0 ± 1.87^aF^	18.0 ± 2.55^aE^	42.0 ± 3.00^aD^	75.0 ± 3.54^aC^	94.0 ± 1.87^aB^	100 ± 0.00^aA^
		4^th^	00 ± 0.0^aG^	5.0 ± 1.58^bF^	14.0 ± 1.87^bE^	31.0 ± 3.67^bD^	63.0 ± 3.39^bC^	85.0 ± 4.18^bB^	100 ± 0.00^aA^
	Co LDH (PP)	2^nd^	1.0 ± 1.0^aG^	10.0 ± 2.24^aF^	22.0 ± 2.00^aE^	43.0 ± 2.00^aD^	81.0 ± 4.30^aC^	95.0 ± 1.58^aB^	100 ± 0.00^aA^
		4^th^	1.0 ± 1.0^aG^	7.0 ± 2.00^bF^	17.0 ± 3.39^bE^	37.0 ± 3.00^bD^	68.0 ± 4.06^bC^	91.0 ± 3.67^bB^	100 ± 0.00^aA^
	Zn LDH (PP)	2^nd^	2.0 ± 1.2^aG^	9.0 ± 1.87^aF^	21.0 ± 1.87^aE^	52.0 ± 5.15^aD^	82.0 ± 2.00^aC^	95.0 ± 2.24^aB^	100 ± 0.00^aA^
		4^th^	2.0 ± 1.2^aG^	6.0 ± 1.87^bF^	16.0 ± 2.92^bE^	36.0 ± 2.92^bD^	66.0 ± 2.45^bC^	89.0 ± 2.45^bB^	100 ± 0.00^aA^
	PP -NE	2^nd^	2.0 ± 1.2^aE^	24.0 ± 3.32^aD^	52.0 ± 6.63^aC^	77.0 ± 4.64^aB^	100 ± 0.00^aA^	100 ± 0.00^aA^	100 ± 0.00^aA^
		4^th^	1.0 ± 1.0^bF^	23.0 ± 2.00^bE^	38.0 ± 4.64^bD^	63.0 ± 6.04^bC^	90.0 ± 2.24^bB^	100 ± 0.00^aA^	100 ± 0.00^aA^
	Free-LDH	2^nd^	00 ± 0.0^aG^	4.0 ± 1.87^aF^	8.0 ± 1.22^aE^	13.0 ± 1.22^aD^	18.0 ± 2.55^aC^	22.0 ± 3.00^aB^	31.0 ± 1.87^aA^
		4^th^	00 ± 0.0^aG^	1.0 ± 1.00^bF^	5.0 ± 2.24^bE^	10.0 ± 2.24^bD^	15.0 ± 3.16^bC^	19.0 ± 2.92^bB^	24.0 ± 2.45^bA^
	Free-NE	2^nd^	00 ± 0.0^aG^	3.0 ± 1.22^aF^	6.0 ± 1.87^aE^	10.0 ± 1.58^aD^	13.0 ± 2.00^aC^	18.0 ± 3.00^aB^	25.0 ± 2.74^aA^
		4^th^	00 ± 0.0^aG^	1.0 ± 1.00^bF^	4.0 ± 2.45^bE^	7.0 ± 2.55^bD^	10.0 ± 3.16^bC^	15.0 ± 2.74^bB^	20.0 ± 2.74^bA^
	Temephos (1 mg/l) *	2^nd^	100 ± 0.00^a^	-	-	-	-	-	-
		4^th^	90.0 ± 2.24^b^	-	-	-	-	-	-

a, b & c: There is no significant difference (P > 0.05) between any two means for the same attribute, within the same column have the same superscript letter; A, B & C: There is no significant difference (P > 0.05) between any two means, within the same row have the same superscript letter (one-way ANOVA, Tukey's range test (P > 0.05); Oil (AJ): Jasmine’s oil blend oil; Oil (PP): Peppermint oil; Co LDH (AJ or PP): Co LDH loaded Jasmine oil blend or Peppermint oils; Zn LDH(AJ or PP): Zn LDH loaded Jasmine's oil blend or Peppermint oils; AJ -NE: Jasmine oil blend nanoemulsion; PP-NE: Peppermint nanoemulsion; Free-LDH: Layered double hydroxides without loading; Free-NE: without oils; * concentration ( ppm); *: the positive control (1 mg/l) was used as recommended dose.

In the present study, both LC_50_ and LC_90_ values PT of L2 were calculated for AJ (412.16 and 846.57 ppm), Co LDH (AJ) (395.24 and 766.90 ppm), Zn LDH (AJ) (396.86 and 790.31 ppm), and AJ-NE (157.07 and 305.73 ppm); whereas the corresponding values of L4 were 488.40 and 1712.64; 446.70 and 1257.65; 477.45 and 1693.64; and 391.69 and 857.16 ppm, respectively. On the other hand, LC_50_ and LC_90_ values were determined PT of L2 with PP (400.85 and 768.84 ppm), Co LDH (PP) (378.39 and 749.53 ppm), Zn LDH (PP) (359.67 and 710.56 ppm), and PP-NE (136.18 and 278.93 ppm), and the corresponding values for L4 were 460.22 and 1625.14; 422.84 and 1110.40; 456.14 and 1483.43; and 346.26 and 763.84, respectively (Table 8).

**Table 8:** The larvicidal effects of Jasmine oil blend and Peppermint oil and their Nano formulations against *Culex pipiens*, 24 h post-treatment.

Oil name	Nano- Formulations	Stage	LC_50_ (Low- Up.)	LC_90_ (Low- Up.)	LC_95_ (Low- Up.)	Chi (Sig)	Slope ± SD	Relative toxicity
Jasmine blend	Oil (AJ)	2^nd^	412.16 (303.39–6638.91)	846.57 (641.43–1355.06)	963.34 (726.33–1569.01)	19.15 (0.002a)	0.003 ± 1.5	1.00
		4^th^	488.40 (140.61–1181.25)	1712.64 (1293.99–3410.18)	2035.15 (1492.11–4198.55)	41.50 (0.000a)	0.86 ± 2.61	1.00
	Co LDH (AJ)	2^nd^	395.24 (272.03–591.44)	766.90 (576.35–1261.06)	872.26 (652.24–1461.20)	20.08 (0.001a)	0.004 ± 1.2	1.10
		4^th^	446.70 (120.19–953.88)	1257.65 (832.09–3338.14)	1478.83 (986.78–4136.99)	72.76 (0.000a)	0.33 ± 1.11	1.10
	Zn LDH (AJ)	2^nd^	396.86 (251.36- 640.34)	790.31 (574.73–1442.21)	901.85 (652.83–1683.10)	25.58 (0.000a)	0.003 ± 0.5	1.10
		4^th^	477.45 (147.28–1101.25)	1693.64 (1203.99–3359.82)	1990.15 (1412.81–4098.51)	51.70 (0.000a)	0.51 ± 9.56	1.02
	AJ-NE	2^nd^	157.07 (118.63–215.15)	306.73 (240.35–456.40)	349.16 (271.69–527.69)	11.64 (0.040a)	0.008 ± 1.0	2.77
		4^th^	391.69 (147.33–755.89)	857.16 (584.36–2189.81)	989.11 (672.09–2632.47)	23.64 (0.000a)	0.003 ± 1.0	1.25
Peppermint	Oil (PP)	2^nd^	400.85 (280.69–590.64)	768.84 (582.15–1237.794)	873.15 (657.813–1431.04)	19.22 (0.002a)	0.004 ± 2.0	1.08
		4^th^	460.22 (121.26–1101.11)	1620.14 (1201.66–3341.24)	2035.15 (1492.11–4198.55)	45.50 (0.000a)	0.72 ± 2.37	1.06
	Co LDH (PP)	2^nd^	378.39 (247.18–593.36)	749.52 (551.55–1314.67)	854.74 (625.94–1531.05)	23.06 (0.000a)	0.0035 ± 0.5	1.15
		4^th^	422.84 (935.85–1182.96)	1110.40 (666.78–7866.34)	1305.31 (801.80–10,080.30)	108.71 (0.000a)	0.24 ± 1.17	1.16
	Zn LDH (PP)	2^nd^	359.67 (199.12–663.44)	710.56 (499.18–1597.62)	820.30 (567.71–1878.98)	34.81 (0.0001a)	0.004 ± 2.00	1.21
		4^th^	456.14 (136.12–1001.55)	1483.43 (1050.19–3214.22)	1789.25 (1241.81–3991.15)	32.74 (0.001a)	0.36 ± 8.83	1.07
	PP-NE	2^nd^	136.18 (97.12–193.12)	278.93 (214.26–437.32)	319.39 (243.60–510.28)	13.59 (0.0180a)	0.008 ± 1.0	3.19
		4^th^	346.26 (80.20–741.78)	763.89 (506.20–2368.85)	882.28 (584.130–2872.94)	27.66 (0.000a)	0.003 ± 1.0	1.41

Since the significance level is less than .050, a heterogeneity factor is used in the calculation of confidence limits; Relative toxicity (folds) = LC50 of the least toxic photosensitizers / LC50 of the tested photosensitizer.

Data on the relative toxicities of the applied materials in this study point out that PP nanoemulsion was the most effective larvicide followed by AJ nanoemulsion. Relative toxicities of the tested larvicides against L2 larvae indicated that AJ-Co, AJ-LDH-ZN, AJ-NE, PP oils, PP-LDH-Co, PP-LDH-Zn, and PP-NE were 1.10, 1.10, 2.77, 1.08, 1.15, 1.21, and 3.19 folds, respectively, as toxic as AJ oil. While the corresponding relative toxicities against L4 were 1.10, 1.02, 1.25, 1.06, 1.16, 1.07, and 1.41 times, respectively (Table 8).

The moderate insecticidal efficacies showed by LDH and their nanoformulations with AJ and PP essential oils compared to the high efficacies of AJ and PP nanoemulsions might be due to the incompatibility of essential oils to the positively charged LDHs, in contrast to the promising results of essential oil nanoemulsion. The low values of the slope resulted from the bioassays PT of L2 indicating different degrees of heterogeneity of larvae and their susceptibility to the applied oils and their nanoformulations. On the other hand, slope values of the toxicity lines of the 4th instar larvae of the tested insects treated with (AJ and PP) and their nanoformulations were recorded as high slope values indicating relative degrees of homogeneity of the tested instar larvae for their toleranct to such oils and their nanoformulations.

In contrast to our findings, a study indicated the larvicidal and adulticidal activity of green tea oil and their LDH Nanoclay against *Cx. pipiens*, in vitro and in the field conditions^84^. The reason for that paradox may be related to the nature of essential oils and nanocarrier, being green tea water is a soluble essential oil makes the nature of active ingredients enriched with polar compounds that may introduce a convenient interpretation of why LDH loaded essential oils, discussed herein, introduced low to moderate insecticidal activities and the data given by GC/MS/MS and GC/MS of low concentrations of active ingredients confirmed the same hypothesis. Using nanoemulsion as a drug carrier ensured high loading capacity and consequently higher concentrations of active ingredients released, which affected the insecticidal positively. The findings of present work regarding high insecticidal activity showed by AJ-NE and PP-NE nanoformulations agreed with several studies reported similar insecticidal efficacy of nanoemulsions^119–122^.

Similar studies based on jasmine oil revealed an extremely strong larvicidal effect against *Aedes aegypti* and *Anopheles stephensi* larvae with LC_50_ = 42.85 and 73.52 ppm, respectively^123^. Our findings were in agreement with other study reported that *Jasminum grandiflorum* essential oil has very strong acaricidal properties against the two-spotted spider mite, *Tetranychus urticae*, with a high-efficiency percentage of 68.50% and a reduction rate of 49.03%^124^.

Some other studies confirmed the effectiveness of oils and their nanoemulsions on insects. Peppermint nanoemulsion recorded a high toxic effect against *Cx. pipiens* (LC_50_ = 43.57 μg/ml) and *Musca domestica* (LC_50_ = 65.13 μg/ml) when compared to peppermint oil and lambdacyhalothrin as larvicides. Nanoformulation was more toxic by 71.46% and 52.0% against *Cx. pipiens* and *M. domestica*, than the crude oil, respectively^125^. Nanoemulsion of peppermint oil against the honeybees, *Apis mellifera*, was highly toxic than the crude oil and their LC_50_ values were 2629.85 and 5471.13 ppm, respectively, after oral treatment, and 4246.84 and 11,895.65 ppm, respectively, after contact treatment^126^. Like our results of PP, the Nanoemulsion of *Mentha piperita* exhibited high toxicity (LC_50_ = 3879.5 ± 16.2 μl a.i. /L) against the cotton aphid, *Aphis gossypii*^127^.

Against the Egyptian strain of *Cx. pipiens* and alike our findings, some other oils induced a similar insecticidal effect and adversely affected the pupation and adult emergence rates with developmental alterations of larvae in Egypt such as *Nigella sativa*, *Allium cepa,* and *Sesamum indicum* oils^49^ as well as fenugreek (*Trigonella foenum-grecum*), earth almond (*Cyperus esculentus*), mustard (*Brassica compestris*), olibanum (*Boswellia serrata*), rocket (*Eruca sativa*), and parsley (*Carum ptroselinum*) oils (LC_50_ = 32.42, 47.17, 71.37, and 83.36, 86.06, and 152.94 ppm, respectively)^50^. Oil resins such as *Commiphora molmol, Araucaria heterophylla, Eucalyptus camaldulensis, Boswellia sacra,* and *Pistacia lentiscus* induced larvicidal effects against *Cx. pipiens* in Egypt^23^. The larvicidal activity of 32 oils (1000 ppm) against the early 4th larvae of *Cx. pipiens* resulted in mortalities 48 h PT (60.0–100% and LT_50_ = 9.67 and 37.64 h for *Thymus vulgaris* and *Sesamum indicum*). The highly effective group of oils induced 95–100 MO%, and included *Anethum graveolens, Allium sativum, Foeniculum vulgare, Camellia sinensis, Salvia officinalis, Nigella sativa, Viola odorata,* and *T. vulgaris.* Such oils induced adulticidal effects as well^128^. Eighteen oils had larvicidal effects against the early 4th larval stage of *Cx. pipiens* inducing 55 to 100 MO%, 24 h PT with 2000 ppm. The highly effective oils, *Azadirachta indica, Lupinus luteus*, *Cyperus alternifolius, Lactuca sativa, Persea Americana,* and *M. alternifolia* induced 95–100 MO% and their LC_50_ values were 588.31, 677.45, 496.96, 611.60, 646.34, 445.28 ppm, respectively; whereas their LC_99_ values were 1601.14, 1331.06, 1953.29, 1667.27, 1342.56, and 1725.94 ppm, respectively^129^.

*Baccharis reticularia* and limonene nanoemulsions were effective against *Ae. aegypti* (LC_50_ = 118.94 g/ml and 81.19 g/ml, respectively)^130^. *Lagenaria siceraria*- synthesized AgNPs were effective against *Cx. pipiens* first to fourth instar larvae and pupae (LC_50_ = 15.2, 18.2, 22.5, 24.7, and 29.4 ppm, respectively) and against *Anopheles pharoensis* (LC_50_ = 11.9, 14.5, 17.7, 19.8, and 23.1 ppm, respectively)^131^**.** Moreover, silver nanoparticles induced mosquito larvicidal effects^24,27,63,64^. Some plant extracts and their nanoformulations effectively controlled the camel tick, *Hyalomma dromedarii*^52,67,132–136^. *Satureja montana* was the most effective acaricide (100% toxicity) against the poultry red mite, *Dermanyssus gallinae*, 48 h PT, while *Thymus vulgaris* had the greatest residual effect (11% toxicity)^59^.

#### Enzymes biochemical assays

In the insect’s body, the increased level of the detoxification enzymes indicates the efficiency of the applied insecticide. Enzymes like Acetylcholinesterase (AChE), carboxylesterase, α-esterases, β-esterases, and glutathione S-transeferase (GST), ensure insecticidal susceptibility. For example, Glutathion S-transferase is one of the most important detoxifying enzymes that protect insect cells from oxidative damage^68^. The resistance of some insects to insecticides is expressed by an elevated GST level^69^.

Regarding enzyme levels, the results of this study indicated a significant increase in phenoloxidase, α and β esterase in the treated groups. Post-treatment of L4 with AJ, PP, AJ-NE, and PP-NE, the phenoloxidase's level reached 545.67, 799.67, 731.00, and 700.00 mO. D. units/min/mg protein, respectively; the levels of α esterase were 9.71, 10.32, 8.81, and 10.55 mg α-naphthol/min/mg protein, respectively; and the levels of β esterase were 3.99, 4.81, 3.75, and 4.39 mg β-naphthol/min/mg protein, respectively (Table 9).

**Table 9:** Effect of jasmine oil blend and Peppermint oil and their Nano formulations on Phenoloxidase and non- Specific Esterase levels in *Culex pipiens.*

Enzyme	Enzymes level (Mean ± SD)				Control Mean ± SD
	Jasmine bled		Peppermint		
	Oil	Nanoemulsion	Oil	Nanoemulsion	
Phenoloxidases (mO.D. units/min/mg protein)	545.67 ± 8.33^b^	731.00 ± 13.23^a^	700.00 ± 10.00^b^	799.67 ± 18.50^b^	669.67 ± 14.57^a^
Alpha esterase (mg α-naphthol/min/mg protein)	9.71 ± 0 .09^a^	10.32 ± 0.12^a^	8.91 ± 0.080^a^	10.55 ± 0.13^b^	8.87 ± 0.061^a^
Beta esterase (mg β-naphthol/min/mg protein)	3.99 ± 0.15^b^	4.81 ± 0.26^b^	3.75 ± 0.13^a^	4.39 ± 0.20^a^	3.99 ± 0 .15^a^

a: means that there is a significant difference between the control and treated group (*p* < 0.05); b: means that there is no significant difference between control and treated groups (*p* > 0.05); NE: nanoemulsion.

Data from this investigation revealed a significant increase in the levels of examined enzymes when compared to the control group. Such an increase comes along with the findings of some other studies reporting a significant increase in amylase, total protein, and lipid levels in adult *A. mellifera* after peppermint and its nanoemulsion treatments^126^. Also, treatment with nano-selenium peppermint increased the activities of phenylalanine ammonia lyase enzyme. Furthermore, the foliar applications of nano-selenium peppermint led to the piling up of soluble phenols and a significant decrease in nitrate levels and peroxidase.

To acquire a satisfactory explanation for the effect of the tested plant oils and their nanoemulsion, a biochemical assay, two enzymes, including phenoloxidase, α and β esterase were evaluated in this study. Phenoloxidase influences the insect immune responses of insects stimulating quinones biosynthesis and other reactive intermediates to remove infesting parasites and pathogens^137^. Most esterases belong to the carboxylesterase family α and β esterase^138^ playing a vital role in detoxification mechanisms and are dominant and remarkable in the metabolism of many kinds of endogenous and exogenous poisonous^139^. Regarding the mode of action**,** some oils have a neurotoxic effect and interfere with the neuromodulator octopamine and gamma-aminobutyric acid, GABA, gated chloride channels^140^ whereas nanoparticles enter the cuticular membrane of mosquito larvae than to their intestine and damage their DNA banding pattern^141^. Also, the essential oil extracted from *Artemisia vulgaris* has outstanding larvicidal action, causing excellent mortality (LC_50_ = 6.87 g/ml) against *Ae. aegypti* larvae because midget cells were injured^142^.

Glutathion S-transferase is one of the most important detoxifying enzymes that protect insect cells from oxidative damage^68^. The resistance of some insects to insecticides is expressed by an elevated GST level^69^. The enzymes responsible for the detoxification and/or metabolism of external toxins (insecticides) are carboxylesterase and α and β esterase enzymes^143^. The treatment of *Cx. pipiens* with some neonicotinoid insecticides caused a potential increase in both carboxylesterase and (α and β) esterase activities^144^.

The suggested activity of nanoformulations against mosquito larvae may be due to their permeation of the exoskeleton to larval cells, where they enclose macromolecules like proteins and DNA, changing their structure and therefore their function^131^. The results of the present study affirm the previous results dealing with the effects of many essential oils and nano-formulations against different mosquito species**.** In this study, the higher toxic effect of the nanoemulsions than the crude essential oil may be because of the small size of the nanoemulsion droplets, which leads to increased surface area and expedites the permeation of nanoemulsion into the insect body^119^. The plant derivative molecules can react with insect body enzymes and hormones and join membranes and cellular constituents, and consequently interpose with the biochemical activity of mosquitoes^145^.

## Conclusions

Recently, novel and eco-friendly control tools to combat vector outbreaks have become an essential demand. Nanotechnology has the potential to expand the spectrum of performance of existing insecticidal effects of essentials, phytocompounds, and other materials by improving their physical, chemical, and biological properties. Increased pesticide effectiveness is achieved by using lower concentrations, reducing the insecticide concentration, and downgrading environmental side effects^61,62,146^.

The data of the present study indicated that PP and AJ, and their nanostructured formulations, had insecticidal activity against *Cx. pipiens* L2 and L4. Remarkably, PP-NE and AJ-NE nanoemulsions revealed promising and potential insecticidal activities. Meanwhile, LDH and their formulations showed efficacies comparable to those results obtained by the crude essential oils.

This study concluded that nanoemulsion was the better delivery system for AJ and PP EOs oils than LDH due to the incompliance polarity between the positively charged LDH carrier and the target essential oils, which are completely immiscible in water and consequently contain very low concentrations of polar nonvolatile active ingredients. Due to the high loading capacities of synthesized NEs, the larvicidal efficiency of nanoemulsions of AJ and PP was significantly increased compared to their crude oils or even the LDH analogy. Besides the highest delivery and chemical stability of the substance, low cost, water dispersal, target action, and low ecological toxicity, these NEs are excellent pesticides. It could be concluded that nanopesticides could lead to a new generation of effective, eco-friendly alternatives that could be applied to controlling mosquito-borne diseases. Field application and ecotoxicological studies of the applied nanoemulsions are recommended as further studies.

## Data Availability

The datasets used and/or analyzed during the current study available from the corresponding author on reasonable request. Correspondence: mohamed.albaz@fsc.bu.edu.eg.
